# Sanshool on The Fingertip Interferes with Vibration Detection in a Rapidly-Adapting (RA) Tactile Channel

**DOI:** 10.1371/journal.pone.0165842

**Published:** 2016-12-09

**Authors:** Scinob Kuroki, Nobuhiro Hagura, Shin’ya Nishida, Patrick Haggard, Junji Watanabe

**Affiliations:** 1 NTT Communication Science Laboratories, NTT Corporation, Kanagawa, Japan; 2 Institute of Cognitive Neuroscience, University College London, London, United Kingdom; 3 Center for Information and Neural Networks (CiNet), National Institute of Communication and Technologies, 1–4 Yamadaoka, Suita City, Osaka, Japan; University of Ottawa, CANADA

## Abstract

An Asian spice, Szechuan pepper (sanshool), is well known for the tingling sensation it induces on the mouth and on the lips. Electrophysiological studies have revealed that its active ingredient can induce firing of mechanoreceptor fibres that typically respond to mechanical vibration. Moreover, a human behavioral study has reported that the perceived frequency of sanshool-induced tingling matches with the preferred frequency range of the tactile rapidly adapting (RA) channel, suggesting the contribution of sanshool-induced RA channel firing to its unique perceptual experience. However, since the RA channel may not be the only channel activated by sanshool, there could be a possibility that the sanshool tingling percept may be caused in whole or in part by other sensory channels. Here, by using a perceptual interference paradigm, we show that the sanshool-induced RA input indeed contributes to the human tactile processing. The absolute detection thresholds for vibrotactile input were measured with and without sanshool application on the fingertip. Sanshool significantly impaired detection of vibrations at 30 Hz (RA channel dominant frequency), but did not impair detection of higher frequency vibrations at 240 Hz (Pacinian-corpuscle (PC) channel dominant frequency) or lower frequency vibrations at 1 Hz (slowly adapting 1 (SA1) channel dominant frequency). These results show that the sanshool induces a peripheral RA channel activation that is relevant for tactile perception. This anomalous activation of RA channels may contribute to the unique tingling experience of sanshool.

## Introduction

Szechuan pepper, a widely used spice in the cuisine of many Asian countries, is well known for the unique tingling and numbing sensation it induces on the tongue and on the lips [1, 2]. This “tingling” is experienced as a tactile phenomenon. The question is how this sensation is elicited without any physical touch, and which aspect of tactile processing it taps into.

Previous studies in mice have shown that the bioactive component of Szechuan pepper, hydroxyl-a-sanshool (hereafter sanshool), elicits chemical events within the receptor membrane and induces burst of neuronal firing on low-threshold rapidly adapting mechanoreceptor fibres that respond to mechanical vibrations [3–6]. Furthermore, Hagura et al. [7] have previously demonstrated that, in humans, the perceived frequency of the sanshool-induced tingling on the lips was 50 ± 2.4 Hz. It is known that the human sensory system processes mechanical input in a frequency dependent manner. The rapidly adapting (RA) afferent channel is sensitive to lower vibration frequencies (peaks around 30Hz), the Pacinian-corpuscle (PC) channel is sensitive to higher frequencies (peaks around 240Hz), and the slowly adapting 1 (SA1) channel is sensitive to slow skin deformation such as sustained pressure [8–10]. Due to the similarity between this RA preferred frequency range and the perceived frequency range of the sanshool-induced tingling experience, Hagura et al suggested that sanshool-induced fibre activation and the low-frequency mechanical (RA) channel input may share the same processing pathway. However, their hypothesis remains elusive, since the conscious perception of stimulus frequency could be generated at a processing stage after information has been combined across channels.

In fact, sanshool does not only activate one specific type of light touch RA channel fibres. Previous neurophysiological studies of dorsal root ganglion neurons in mice have shown that a range of tactile fibres are influenced by sanshool. Activations of rapid-adapting Aβ (RA), rapid-adapting Aδ (D-hair), and slowly-conducting C fibres were reported, while inhibition of slow-adapting Aβ (SA), slow-adapting Aδ (mechanosensory nociceptors), and fast-conducting C fibres were also reported [5, 11, 12]. Therefore, the sanshool experience could potentially depend on the combined activation of tactile channels other than the RA channel. Indeed, it is also logically possible that the sanshool-induced RA channel input does not contribute to the human tactile experience at all.

In this study, we elucidate whether the RA channel activation is indeed a major contributor to the human tactile processing in sanshool-induced sensation compared to other mechanical channel inputs. We examined if the application of sanshool on the fingertip interfere with the detection of 30Hz, 240Hz and 1Hz vibrotactile inputs that preferentially stimulate RA, PC and SA1 mechanoreceptor channels, respectively. It has been shown that the masking/adaptation effect on tactile signal detection reveals the vibro-frequency tunings similar to those of the mechanoreceptor channels responsible for signal detection [8, 13–21]. This indicates that the interference/masking effect dominantly reflects signal interactions within each individual mechanoreceptor channel, and therefore the psychophysical method should provide direct and objective evidence about the contribution of individual peripheral frequency channels during sanshool-induced tingling-like sensation, in addition to the measurement of subjective perceptual frequency similarity [7]. We did not test sanshool effect on SA2 channel, since this channel is not vibration sensitive and therefore is irrelevant for vibratory stimuli [8]. It should be noted that, since our interest was the contribution of RA mechanoicalrecpetor channel to the sanshool-induced sensation, the present paradigm evaluates the contribution of mechanoreceptors during the application of sanshool, but not the contribution of other non-mechanical channels.

If the RA fibre activation is indeed the major sanshool related mechanoreceptor input processed in the brain, detection threshold of RA vibrotactile input should increase with application of sanshool due to the increased activation (noise level) of that pathway. In this case, the detection of vibration in other frequency ranges, carried by other channels, will be unchanged. If the sanshool-induced firing of the mechanical fibres does not impact tactile perceptual experience, detection thresholds for all of the input channels will be unaffected. We apply the same logic also to other frequency channels, to see if their perceptual processing is also affected by sanshool.

## Results

### Sanshool tingling on the fingers

Previous studies of sanshool-induced tingle used the lips as the stimulation site, because of the ease of inducing the tingle sensation there. In contrast, the vast majority of psychophysical and neurophysiological research on touch has used the fingertip as stimulation site [22–26]. Since the PC channel mechanoreceptor is absent in orofacial skin [27–29], our aim of testing multiple channels suggested using the fingertips, rather than the lips. We therefore first confirmed that the tingling sensation by sanshool could be also reliably elicited on the finger, not only on the lips as it has been shown previously [7].

A chemical extract of hydroxyl-alpha-sanshool (ZANTHALENE, Indena) was applied on twelve participants’ left index or middle finger (target finger), which was counterbalanced across participants. The other finger was designated as a control finger, and stimulated with ethanol instead of sanshool. Since the skin of the finger is thicker than that of the lips, we asked participants to scratch the skin surface of both fingers with a lancet (softclix, ACCU-CHEK, USA), to allow effective penetration of sanshool.

Participants were asked whether they felt any difference in tactile sensation between the target and the control finger. The occurrence of “tingling sensation” was explicitly mentioned. 83.3% of participants (10 out of 12) reported reliable tingling-like sensation only on the target finger, of the remains of participants with one reported no distinct sensations on either finger, while one reported a weak tingling-like sensation on both fingers. The onset latency of the tingling sensation from when the sanshool was applied, and also the perceived intensity of the sensation, varied across participants. Four participants reported strong tingling sensation immediately following the application. Two participants reported a clear but weak tingling sensation immediately after the application. Four participants reported a sensation that emerged gradually, and then became strong within a period of 30 minutes.

Two of our twelve participants never reported any tingling sensation, even after 30 minutes from the sanshool application. Since all of the participants reported the tingling sensation on the lips in the previous study [7], this result may be due to the individual differences in the skin thickness on the finger; for these two participants, the sanshool may have not reached the dermic layer to activate the receptors/fibres. Therefore, these two participants were excluded from the further experiments.

### Sanshool effect on detection of RA preferred vibration (30Hz)

**Fig 1:**
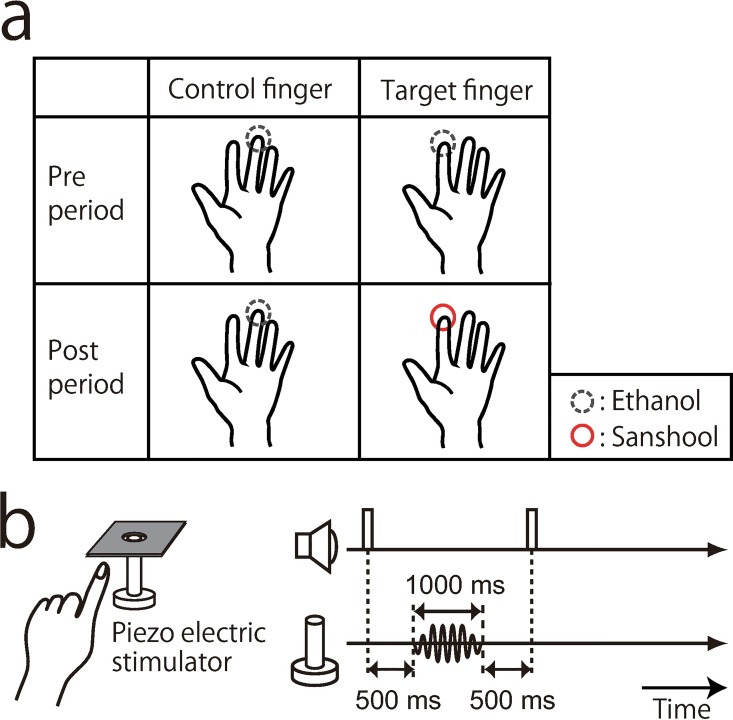
Experimental procedure and setup. (a) Detection thresholds of vibration were measured in four conditions (2 fingers x 2 periods). In pre-period, 40% ethanol was applied to both fingers. In the post-period, the sanshool liquid was applied to the target finger and 40% ethanol was applied to the control finger. The assignment of the target finger was counterbalanced across participants. (b) Experimental setup and the sequence of a trial. Participants positioned their left index or middle fingertip on the piezo electric stimulator. The finger itself was supported by a board surrounding the stimulator. Beeps indicated the start and end of two stimulus presentation intervals, and vibration with random intensity (amplitude) was presented for 1000ms in the first or second interval. Participants were asked to report in which of the two periods the vibration was presented.

In this experiment, we measured detection threshold of 30Hz vibration with or without sanshool on the fingers. This frequency is primarily detected by the RA channel, which was hypothesized to be the main mediator of sanshool-induced tingling sensation. Once the tingling sensation begins after the application of sanshool, it cannot be washed out, but only decays slowly over time. This makes it practically difficult to counter-balance the order of trials with and without sanshool trials. We therefore favored a design with a control finger, rather than control trials. Further, we measured detection thresholds before and after sanshool application on one finger (Fig 1A). For the control finger, ethanol was applied instead of sanshool. In this design, a change in detection threshold of the target finger (pre target vs. post target) represents a combination of sanshool effect and various temporal effects (e.g., learning, fatigue, condition order etc.), whereas that of the control finger represents only the temporal effect. Therefore, differences in threshold shift between the target and the control finger were used as an estimate of sanshool-induced changes in the target frequency detection.

**Fig 2:**
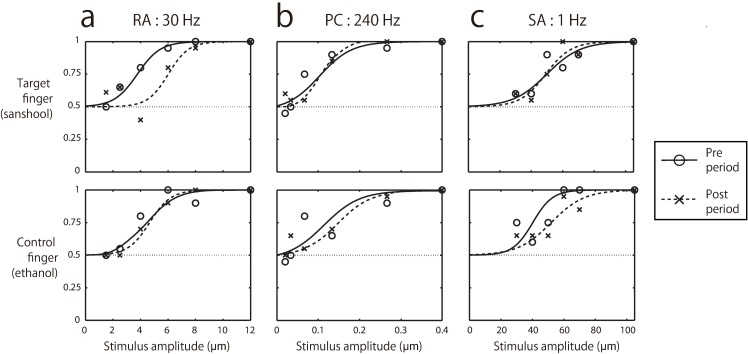
Typical examples of participants’ detection responses. Panels A, B, and C correspond to the RA-, PC-, and SA-preferred vibration detection conditions, respectively. Each point was calculated from 20 repetitions. Each psychometric function was fit by a cumulative Gaussian function.

In each trial, one 30Hz vibration stimulus was presented for 1000ms at one of two time periods defined by a series beeps (Fig 1B). Participants were asked to indicate during which of the two periods a mechanical vibration was presented to the fingertip (2 Interval forced choice (2IFC)). The intensity (amplitude) of the vibration varied randomly across trials (method of constant stimuli). Detection threshold was defined as the vibration amplitude corresponding to participants’ 75% correct detection of the stimulus estimated from the cumulative Gaussian function fitted to the proportion of correct responses across different stimulus amplitudes (Fig 2A). The averaged thresholds were 5.0 ± 1.6 μm for the target finger-pre period, 6.0 ± 1.1 μm for the target finger-post period; 5.1 ± 1.8 μm for the control finger-pre period, and 5.2 ± 1.4 μm for the control finger-post period. We performed a two-way repeated measures analysis of variance (ANOVA) with fingers (target and control) and periods (pre- and post-) as factors and the detection threshold values as the dependent variable. There were no significant effects of fingers [F(1,9) = 0.83, p = 0.39, η2 = 0.0094] nor periods [F(1,9) = 2.8, p = 0.13, η2 = 0.032], but the interaction effect between these factors was significant [F(1,9) = 6.1, p = 0.034, η2 = 0.023]. The post-hoc comparison revealed a significant difference between pre- and post-periods of the target finger [F(1,9) = 7.1, p<0.02 by Ryan’s method] but not for the non-target finger [F(1,9) = 0.051, p>0.8], indicating that the sanshool-induced threshold difference cannot be explained by the initial threshold bias between the fingers before the sanshool application. This is consistent with the hypothesis that sanshool effectively added noise to the RA channel, impairing the signal detection sensitivity within that channel.

### Sanshool effect on detection of PC-preferred (240Hz) and SA1-preferred (1Hz) vibration

**Fig 3:**
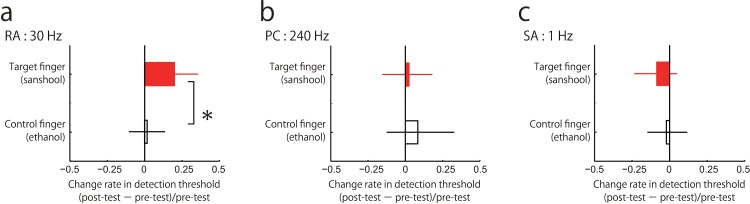
Sanshool effect on vibration detection. Change rate in vibration detection threshold in the post compared to the pre period, for the test and the control finger. Positive values indicate an increased threshold (lower sensitivity) relative to the pre-test period. Error bars represent 95% confidence intervals, which were calculated by the boot strapping method [41]. * < 0.05

To test the frequency selectivity of this sanshool-induced noise, we tested whether the same sanshool effect of threshold elevation can be also observed for detecting higher frequency input (PC-preferred frequency range; 240Hz) or slow skin deformation (SA1-preferred frequency range; 1Hz). We independently examined the detection threshold for each frequency since each channel has different noise distribution. When the change in PC detection threshold was examined using 240Hz vibration, we did not find any threshold elevation nor significant effect of sanshool. The averaged thresholds of PC-preferred frequency range were 0.22 ± 0.29 μm for the target finger-pre period, 0.23 ± 0.25 μm for the target finger-post period; 0.20 ± 0.15 μm for the control finger-pre period, and 0.22 ± 0.20 μm for the control finger-post period (Fig 3B). We confirmed by ANOVA that there is no significant difference in the detection threshold across conditions and fingers. [F(1,9) = 0.088, p = 0.77, η2 = 0.00091 for fingers; F(1,9) = 0.46, p = 0.52, η2 = 0.00058 for periods; F(1,9) = 0.091, p = 0.77, η2 = 0.00018 for interaction].We thus found no evidence for the hypothesis that sanshool activates the PC channel.

Mechanoreceptors responding to pressure (SA1 channel receptors; Merkel cells) and also RA channel receptors (Meissner corpuscles) are located in the superficial part of the skin [30, 31]. In contrast, the PC channel receptors (Pacinian corpuscles) are located in a deeper layer. Thus, the response of the different channels to sanshool might depend on effective stimulation due to penetration of the skin, rather than on any channel-specific molecular mechanism. If distance from the skin were the main determinant of sanshool-induced responding, detection of slow pressure change (1Hz) should also be affected by sanshool. However, we found no evidence that detection of 1Hz vibration was influenced by sanshool. The averaged thresholds of SA1-preferred frequency range were 48 ± 22 μm for the target finger-pre period, 44 ± 19 μm for the target finger-post period; 50 ± 16 μm for the control finger-pre period, and 49 ± 12 μm for the control finger-post period (Fig 3C). Again, we confirmed that there was no significant difference in detection threshold across conditions and fingers [F(1,9) = 0.22, p = 0.65, η2 = 0.0098 for fingers; F(1,9) = 0.59, p = 0.46, η2 = 0.0058 for periods; F(1,9) = 1.6, p = 0.23, η2 = 0.0021 for interaction].

Taken all together, the results show that the sanshool application significantly impaired the detection threshold only when low frequency RA range vibration was presented.

## Discussion

In this study, we showed that the application of sanshool on the finger interferes with perceptual processing of RA-preferred frequency range mechanical input (30Hz vibration) on that finger. On the other hand, we could not find any evidence of interference for the processing of the PC-preferred (240Hz vibration) or for the processing of the SA1-preferred input (1Hz vibration). Consistent with the previous neurophysiological and behavioural studies, our result indicates that, among the three mechanical tactile channels, RA channel is the major channel activated by sanshool, and which impacts on the further tactile information processing.

We used a selective interference/masking approach to investigate sanshool effects on mechanoreceptor-related perception. The masking effect on detection task is vibro-frequency selective, thus, it has been suggested that the signal interaction during masking is happening at the level of within channel processing [8, 13–15, 18, 19, 21]. Also, it has been shown in humans that a single microstimulation pulse to a single RA afferent fibre can induce a light touch sensation [32]. That is, if sanshool were to increase spiking activity in RA afferents, perceptible sensation might be expected. Therefore, we believe that the background firing level within the RA channel was increased by sanshool. The sanshool-induced firing could be considered as an increased level of neural noise in which the small additional signal due to mechanical vibration must be detected. We suggest that the sanshool-induced increased noise level affected detection of vibrotactile input in the preferred range of the RA channel. Moreover, we found no evidence for such interference effects for the other frequency range inputs (PC, SA1). This may suggest that the RA channel is the most sensitive among the set of mechanical channels activated by sanshool. This result corroborate with the neurophysiological findings that the RA fibres are more prominently active than the SA1 fibres [5, 12]. However, note that, effect of sanshool might depend on the location where the sanshool has been penetrated (i.e., skin thickness) since Meissner's corpuscles associated with the RA channel are the receptor located most superficially. Although the location of Merkel cells associated with the SA1 channel are close to that of Meissner’s corpuscles, Pacinian corpuscles associated with the PC channel are located much deeper in the skin. Also, inevitable variability in the scratching process might cause differences across participants in the degree of penetration of sanshool, despite our efforts to control this aspect. Thus, in our study, the sanshool may have not reached to the relevant receptors for the case of PCs, leading to a weaker effective stimulation. Subcutaneous sanshool injections in future investigations could, in principle, resolve this point. In contrast to the interference/masking effect, it is known that detection of a subthreshold tactile signal is enhanced by the presence of random energy fluctuations, due to stochastic resonance [33]. Whether this enhancement effect occurs between sanshool and vibration perception is an intriguing open question, which could be tested with more systematic control of intensity of sanshool stimulation.

Could some non-RA-related effect of sanshool be responsible for the effect we found on RA-mediated vibration detection? This possibility is difficult to exclude conclusively. Clearly, sanshool has effects on several receptors and fibre types, including mechanoreceptive, thermoceptive and nociceptive fibres by activating TRPV1 and TRPA1 [34, 35] or by inhibiting KCNK channels [4, 5]. Also, sanshool or the scratching process could have induced an inflammation of the skin, which can influence the sensitivity to mechanical sensation. In principle, sanshool-induced activity in these channels could potentially interfere with 30 Hz vibration detection at some late, post-channel stage, perhaps due to a non-specific distracting effect. However, we consider this hypothesis unlikely for several reasons. First, a non-specific distracting effect should also influence detection of 1 Hz and 240 Hz vibrations, yet no effect was found. Second, inflammation is known for sensitizing effect on mechanical sensation [36], though this effect would operate in the opposite direction to our finding that detection threshold for mechanical input was increased by sanshool. While a contribution of thermoceptive effects of sanshool also cannot be ruled out, detection of RA-mediated vibration was reported to be largely independent of skin temperature, and thus of thermoceptive afferent activity [37]. Finally, numerous psychophysical and neurophysiological studies confirm that frequency-specific masking/interference effects are readily described by models in which masking occurs independently within each of a number of frequency-specific channels [13–21, 38, 39], with only minimal general frequency non-selective masking effects. Thus, there is clear neurophysiological evidence that sanshool-induced masking/interference could occur within the RA-selective channel, consistent with our psychophysical findings. While the hypothesis of sanshool-induced interference at other, post-channel processing stages cannot be conclusively ruled out, this hypothesis requires additional ad hoc assumptions, for which evidence is so far lacking.

Our experiments were conducted only on the glabrous skin. While animal neurophysiological studies have reported activation of the RA afferent in the hairy skin by application of sanshool [5], it is intriguing open question whether sanshool also interferes the low-frequency detection on the hairy skin as well as that on the glabrous skin.

The aim of the present study is to investigate the impact of sanshool-induced RA activation in the human tactile processing. For this purpose, we used the vibrotactile detection task, traditionally used for studying tactile mechanical channels. Therefore, our finding that the RA channel processing is influenced by sanshool cannot shed light on whether non-mechanical channels, such as nociceptive or thermal fibres, may also contribute to the “mechanical” tingling perception the sanshool elicits. The nature of the unusual “tingling” experience by sanshool cannot be fully understood only from our study.

In conclusion, using a behavioral interference paradigm, we showed that the sanshool-induced RA channel activation contribute to the human tactile processing. This indicates that the unique tactile experience elicited by sanshool involves activation of RA mechanical channel input. Many studies of natural products (chili pepper, mint, mustard oil, etc.) have substantially contributed to the understanding of selectivity and coding principles of the afferent sensory systems underlying pain and temperature perception [40]. Likewise, sanshool could be a useful scientific tool for investigating the relation between specific afferent signals and the resulting perception in the mechano-tactile system, as well as a tool to “flavor” the tactile experiences when used together with the other physical tactile inputs.

## Methods

### Participants

12 (3 females) right-handed volunteers (including two authors), aged from 22 to 38 years (mean = 29.5, sd = 4.9) participated in all of the three experiments. They gave their informed consent before the start of the experiment. Volunteers were unaware of the purpose of the experiments. Ethical approval for this study was obtained from the NTT Communication Science Laboratories Ethical Committee. The experiments were conducted in accordance with the principles of the Helsinki Declaration. Written informed consent was obtained from all participants.

### Apparatus

Tactile stimuli were delivered to the finger pad using a gypsum probe with a 12-mm tip diameter, which was driven by a piezoelectric actuator (MU-120AD12, MESS-TEK, Japan). The resonance frequency of the actuator was 6 kHz, the maximum load was 1200 N, and the maximum pulling force of 200N. We used a position control method to produce vibrations so that the piezoelectric actuator could accurately produce commanded displacement with a tolerance of few nanometers. We confirmed that the movement of the actuator, measured using a laser displacement meter (Keyence LC-2400, JAPAN) without finger contact, do not include any unintended vibration at harmonic frequencies. Due to the large output force and roughly flat frequency response, the movement of this actuator was accurate irrespective of the force from the participant’s finger within the frequency range we used.

A participant sat at a table and placed the left index or middle finger on a stimulator through a hole in a metal board (Fig 1A). The actuator vertically deformed the skin through a hole in a metal board, which prevented the vibration from spreading across the surface of the skin. The diameters of the stimulator and the hole were 12.0 and 14.0 mm, respectively. The stimulator was in contact with the finger throughout the experiment. The duration of the vibration was 1000ms, and its waveform was modulated with 20-Hz raised cosine window at onset and offset. Participants made responses by clicking a mouse with their right hand. They performed experiments with their eyes open to maintain their arousal level but they could not see the vibration of the stimulator. White noise was continuously presented from the headphones throughout the experiment, to mask any subtle sound made by the tactile stimulation. Auditory cues were also presented from the headphones.

Chemical extract of hydroxyl-alpha-sanshool (ZANTHALENE, Indena) was used as chemical stimuli. This is a supercritical CO2 extract from the Zanthoxylum bungeanum, which is standardized in alkylamides. The concentration is almost 100%, and we apply to the participants’ finger as much as we can to induce reliable tingling sensation on the finger. Sanshool reliably produces “tingling” sensation when applied on the lips [7]. Since the skin of the finger is thicker than that of the lips, we asked participants to scratch the surface skin of their fingers with a lancet (softclix, ACCU-CHEK, USA). We explicitly inhibited them from pricking the skin to prevent bleeding. They made scratch over the finger pads of the index and middle fingers of their left hand. Two participants did not report any tingling sensation even after 30 minutes from the sanshool application. Since there could be a possibility that the sanshool has not reached the dermis to activate the receptors for these participants, data from these two participants were excluded from the analysis.

Sanshool we used has strong pungent odor. However, we believe this odor was not a critical factor that affect our experiment, since the participants already knew which finger the sanshool has been applied due to the tingling sensation occurring only on the sanshool applied finger, regardless of the smell.

### Procedure

Participants performed 2 interval forced choice (2IFC) task for detecting the vibration on the finger. Left index and middle finger was used. One of the fingers was assigned as target finger, and the other as control finger. The assignment of target and control from the two fingers was counterbalanced across participants. We used vibration of 30Hz, 240Hz, and 1Hz as the stimulus frequency, and each frequency was tested on a different block. The detection task was performed for two periods on each stimulus frequency; before application of sanshool on the target finger (pre-period) and after the application (post-period).

At the start of the experiment, participants were asked to clean their target and control fingers. In pre-period, 40% ethanol was applied to all over the pads of both fingers. In the post-period, the sanshool liquid was applied similarly to the target finger and 40% ethanol was applied to the control finger. The post-period test started after participant had reported a clear tingling sensation on the sanshool applied finger. Sanshool liquid or the ethanol was topped up to the relevant finger before each block.

A trial consisted of two intervals of 2000ms, each starting with a beep sound. The end of the second period was not defined by the beep. Instead, we informed participants that first and second period have the same duration. A 1000ms vibration was presented only once in a trial, either in the first or the second interval (500ms after the beep). For each stimulus frequency, five or six different levels of the stimulus intensity between 0.25 μm to 100 μm was prepared, which varied from the sub-threshold to supra-threshold level based on a preliminary experiment performed individually before the main experiment. Participants made a binary response as to whether the stimulus was presented after the first beep or the second beep. Auditory cues from actuator were prevented by earplugs and by the pink noise from the headphones. Each participant performed 2 fingers x 2 period x 3 frequency x 5/6 intensities x 20 repetitions. The timing and the intensity of the stimulus was randomized across trials within a block. The stimulated finger was fixed during a block, and randomly changed between blocks. One block is consisted of 50 or 60 trials.

### Analysis

To estimate the detection threshold of each condition (finger (target, control; 2) x period (pre, post; 2)) for each participant, proportion of correct responses were plotter against the stimulus intensity. Then, a cumulative Gaussian function was fitted to the data by using maximum likelihood estimation, with the mean and standard deviation as free parameters. We fixed the lower asymptote to 0.5 and the upper asymptote to 1 since the task was two interval forced choice. From this estimated function, stimulus intensity with 75% correct response was defined as the detection threshold value of each condition.

The effect of sanshool on detection threshold of RA-preferred vibration (30Hz) was assessed by comparing the difference in threshold between the pre- and post- condition between the target and the control finger using paired t-test. Note that threshold shift was normalized by average of pre-period threshold and shown in change rate. We applied the same procedure with different vibration amplitudes to higher frequency input (PC channel; 240Hz; 0.01μm ~1μm) or slow skin deformation (SA1 channel; 1Hz; 20μm ~70μm).
